# Early Diagnosis of Acute Rejection and Acute Tubular Necrosis After Kidney Transplantation Using Magnetic Resonance Imaging: Evaluation of the Diffusion-Weighted Imaging Method

**DOI:** 10.7759/cureus.82879

**Published:** 2025-04-23

**Authors:** Hasan Yasar, Volkan Sayur, Ebubekir Korucuk, Bekir Cetin, Bartu Cetin, Egemen Ozturk, Berk Goktepe, Taylan O Sezer

**Affiliations:** 1 Department of General Surgery, Manisa State Hospital, Manisa, TUR; 2 Department of General Surgery, Ege University Hospital, Izmir, TUR; 3 Department of Radiology, Ege University Hospital, Izmir, TUR

**Keywords:** acute rejection, acute tubular necrosis, acute tubular necrosis (atn), diffusion-weighted mri, diffusion-weighted mri (dwi), early diagnosis, kidney transplantation, renal transplantation

## Abstract

Introduction

Complications such as acute rejection (AR) and acute tubular necrosis (ATN) following kidney transplantation can adversely affect graft function, complicating the treatment process and endangering patient health. Diffusion-weighted magnetic resonance imaging (DWI) has emerged as a promising imaging modality for detecting microscopic changes in renal tissue, particularly those affecting cellular structures. This study aims to evaluate the potential of DWI in detecting AR and ATN in kidney transplant patients.

Methods

A total of 24 patients who underwent kidney transplantation at the Department of Organ Transplantation, Ege University Faculty of Medicine, between January 2010 and December 2019 were included in the study. Fourteen patients with AR or ATN formed the study group, while the remaining 10 patients, who did not develop complications, constituted the control group. All patients underwent DWI using a 3 Tesla magnetic resonance imaging (MRI) device, and apparent diffusion coefficient (ADC) values were measured at different levels of the renal cortex and medulla.

Results

When evaluating the mean ADC values (×10⁻³ mm²/second) measured from the upper, middle, and lower poles of the kidneys, significantly lower values were observed in the study group compared to the control group. Statistically significant differences were found in all regions (p < 0.05).

Conclusion

In conclusion, this study highlights the potential utility of DWI as a non-invasive tool for assessing renal allograft function and detecting early graft injury in kidney transplant recipients.

## Introduction

Kidney transplantation is a widely adopted treatment method for patients with end-stage renal disease, significantly improving their quality of life [1]. However, complications such as acute rejection (AR) and acute tubular necrosis (ATN) following kidney transplantation can adversely affect graft function, complicating the treatment process and endangering patient health [2]. The early detection of these complications is crucial for enhancing treatment strategies and patient outcomes [3].

Current clinical approaches primarily rely on biochemical parameters and histological examinations. However, these methods may sometimes be limited in detecting early stages of complications, potentially leading to missed opportunities for timely intervention [4].

In recent years, diffusion-weighted magnetic resonance imaging (DWI) has gained prominence as a promising imaging modality for detecting microscopic changes in renal tissue, particularly those affecting cellular structures [5]. DWI measures the random movement of water molecules in biological tissues, providing critical information about tissue microstructure [6]. The diffusion rate of these water molecules provides critical insights into tissue integrity and microstructural alterations. The apparent diffusion coefficient (ADC) quantifies water molecule mobility and serves as an indicator of structural changes in renal tissue. ADC is calculated using the negative natural logarithm of the ratio between signal intensities obtained with and without diffusion weighting, scaled by the b value (diffusion sensitivity factor). The formula is expressed as follows: ADC = -ln(S1 / S0) / b, where ADC is given in mm²/second, b represents the diffusion weighting factor, S0 is the signal intensity with no diffusion gradient applied (b = 0), and S1 is the signal intensity measured after applying the diffusion gradient [7]. In cases of AR and ATN following kidney transplantation, changes in ADC values can aid in the early detection of tissue damage [8].

Detecting such microscopic changes in patients with kidney transplants, especially in the early post-transplant period, is of great importance [9]. Traditional imaging techniques often provide limited information in this context, whereas DWI offers detailed and non-invasive data that can improve diagnostic accuracy and facilitate the prompt management of early complications [10].

This study seeks to compare ADC values between patients experiencing AR or ATN and those without such complications using DWI. It seeks to assess the potential of DWI as a diagnostic tool for the early detection of complications and for informing treatment strategies in patients with a kidney transplant.

## Materials and methods

Patient selection and data collection

This prospective study included 24 patients who underwent either living donor or cadaveric kidney transplantation at the Department of Organ Transplantation, Ege University Faculty of Medicine, between January 2010 and December 2019. Among them, 14 patients exhibited signs of AR or ATN and were classified as the study group, while the remaining 10 patients, who did not develop complications, formed the control group.

The inclusion criteria for the study consisted of patients aged between 18 and 70 years, with no contraindications, and who had developed AR or ATN. The exclusion criteria included patients with magnetic resonance imaging (MRI) contraindications, those with renal dysfunction due to conditions such as infection or ureteral obstruction, and those with diseases such as hypoglobulinemia, thalassemia, sickle cell anemia, heart failure, or cardiomyopathy.

Panel reactive antibody (PRA) levels were measured to assess the degree of immunologic sensitization in all transplant recipients. On the day of imaging, serum urea and creatinine levels were measured for all patients. The estimated glomerular filtration rate (eGFR), an important indicator of renal function, was calculated using the Modification of Diet in Renal Disease (MDRD) formula. This formula estimates eGFR as follows: eGFR = 175 × (serum creatinine)^-1.154^ × (age)^-0.203^ × 0.742 (if a woman).

The result is expressed in milliliters per minute per 1.73 square meters (mL/minute/1.73 m²), with serum creatinine measured in mg/dL. The MDRD formula allows for a standardized assessment of kidney function by accounting for age, gender, and serum creatinine levels. In addition, urinary protein level was also evaluated as a biochemical marker in the assessment of kidney function.

Prior to the procedure, all patients were thoroughly informed, and informed consent forms were provided and read by the participants. Any questions regarding the procedure were answered in detail. All patients were hospitalized at the time and were selected randomly.

All patients underwent DWI using a 3 Tesla MRI scanner. The imaging protocol utilized a single-shot echo-planar imaging sequence with b-values of 50, 400, and 800 mm²/second. The MRI acquisition time was 11 minutes and 56 seconds. ADC values were measured from circular regions of interest (ROIs) placed in both the cortical and medullary areas of the upper, middle, and lower poles of the transplanted kidneys. All measurements were performed by an experienced radiologist blinded to the patients’ clinical status, and the average of three measurements from each region was used for statistical analysis.

Statistical analysis

Statistical analyses were conducted using the SPSS software (version 23.0; IBM Corp., Armonk, NY). Descriptive statistics were reported as frequencies and percentages for categorical variables and as means ± standard deviations for continuous variables. Group comparisons were performed using one-way analysis of variance (ANOVA) for normally distributed variables, while the Kruskal-Wallis test was applied for non-normally distributed data. Associations between categorical variables were assessed using the chi-square test or Fisher’s exact test, where appropriate. Independent samples t-tests were used for pairwise comparisons. A two-tailed p-value of <0.05 was considered indicative of statistical significance.

Ethical approval

The study was conducted in accordance with the tenets of the Declaration of Helsinki, and ethical approval was granted by the Ethics Committee of Ege University Faculty of Medicine with document number 16-12.1/49.

## Results

Among the kidney transplant recipients included in the study, the mean age was 44.6 ± 13.65 years in the study group and 41.8 ± 18.3 years in the control group. The study group consisted of seven men (50%) and seven women (50%), while the control group included three men (30%) and seven women (70%). Regarding donor types, in the study group, seven individuals (50%) received kidneys from living donors and seven (50%) from cadaveric donors. In the control group, four individuals (40%) received grafts from living donors and six (60%) from cadaveric donors (Table 1).

**Table 1:** Descriptive data of patients SD: standard deviation

Parameters	Study Group (n = 14)	Control Group (n = 10)	Total (n = 24)
Mean Age ± SD	44.6 ± 13.65	41.8 ± 18.3	43.4 ± 15.5
Female (%)	7 (50.0)	7 (70.0)	14 (58.3)
Male (%)	7 (50.0)	3 (30.0)	10 (41.7)
Living Donor (%)	7 (50.0)	4 (40.0)	11 (45.8)
Cadaveric Donor (%)	7 (50.0)	6 (60.0)	13 (54.2)

The mean eGFR of the 14 patients in the study group was found to be 23.31 ± 13.6, whereas the mean eGFR of the patients in the control group was determined to be 65.97 ± 12.3. The eGFR was found to be significantly higher in the control group (p = 0.003). When urinary protein levels were analyzed, the mean value was 63.1 ± 51.7 mg/dL in the study group and 10.3 ± 3.05 mg/dL in the control group. This difference was statistically significant and showed a strong correlation (p = 0.001). The analysis of PRA data revealed that nine individuals (64.3%) in the study group and four individuals (40.0%) in the control group were PRA-positive (Table 2).

**Table 2:** Laboratory results and the comparison of patients SD, standard deviation; PRA, panel reactive antibody; eGFR, estimated glomerular filtration rate

Laboratory Results	Study Group (n = 14)	Control Group (n = 10)	p
eGFR ± SD (mL/minute/1.73 m²)	23.31 ± 13.6	65.97 ± 12.3	0.003
Urine Protein ± SD (mg/dL)	63.1 ± 51.7	10.3 ± 3.05	0.001
PRA-Positive (%)	9 (64.3)	4 (40.0)	0.652

When evaluating the mean ADC values (×10⁻³ mm²/second) measured from the upper, middle, and lower poles of the kidneys, significantly lower values were observed in the study group compared to the control group. Statistically significant differences were found in all regions (p < 0.05). The ADC values by region and their comparison are presented in Table 3.

**Table 3:** The mean ADC values (×10⁻³ mm²/second) measured from the upper, middle, and lower poles of the kidneys SD, standard deviation; ADC, apparent diffusion coefficient

Region of the Kidney	Study Group (n = 14)	Control Group (n = 10)	p
Cortex Upper Pole ± SD	1.77 ± 0.21	1.96 ± 0.14	0.024
Cortex Midsection ± SD	1.73 ± 0.31	1.98 ± 0.31	0.041
Cortex Lower Pole ± SD	1.68 ± 0.24	1.91 ± 0.12	0.009
Medulla Upper Pole ± SD	1.63 ± 0.17	1.84 ± 0.14	0.013
Medulla Midsection ± SD	1.61 ± 0.29	1.76 ± 0.18	0.024
Medulla Lower Pole ± SD	1.57 ± 0.25	1.76 ± 0.12	0.003

In the study group, the relationship between ADC values in the upper, middle, and lower poles and the presence of AR or ATN, as preliminarily diagnosed based on clinical judgement, was evaluated, and a statistically significant difference was observed in all regions except for the upper pole of the cortex and medulla (Table 4).

**Table 4:** Comparison of mean ADC values (×10⁻³ mm²/second) measured from the upper, middle, and lower poles of the kidneys in the study group, based on the clinician’s judgement SD, standard deviation; ATN, acute tubular necrosis; AR, acute rejection; ADC, apparent diffusion coefficient

Regions of the Kidney	Mean ADC Values Within Clinical Status (×10⁻³ mm²/second)		p
	ATN (n = 7)	AR (n = 7)	
Cortex Upper Pole ± SD	1.81 ± 0.29	1.74 ± 0.11	0.949
Cortex Midsection ± SD	1.81 ± 0.23	1.65 ± 0.36	0.018
Cortex Lower Pole ± SD	1.77 ± 0.13	1.60 ± 0.29	0.027
Medulla Upper Pole ± SD	1.61 ± 0.21	1.65 ± 0.16	0.133
Medulla Midsection ± SD	1.69 ± 0.25	1.52 ± 0.32	0.022
Medulla Lower Pole ± SD	1.67 ± 0.15	1.47 ± 0.31	0.035

## Discussion

Kidney transplantation is the most effective and well-established treatment modality for end-stage renal disease, with one-year graft survival rates reaching up to 97% [11]. Although advancements in surgical techniques through accumulated clinical experience and significant progress in immunosuppressive therapies have improved transplant success rates, AR and ATN remain major early complications that continue to adversely affect graft outcomes [12].

If not promptly diagnosed and treated, AR can lead to severe health complications and significantly increase the risk of developing chronic rejection. Although biopsy remains the gold standard for the diagnosis of AR, increasing interest has emerged in identifying non-invasive diagnostic methods that can detect renal injury due to AR before the onset of clinical symptoms. Many biomarkers have been proposed, and research in this area is actively ongoing [13,14].

DWI is a non-invasive technique that enables the evaluation of structural and functional changes in the kidneys and the assessment of microscopic alterations in the renal parenchyma through ADC measurements [15,16]. Many studies in the literature have investigated the utility of DWI in diagnosing kidney failure and AR following kidney transplantation. In this study, we aimed to evaluate the effectiveness of DWI in diagnosing acute allograft kidney injury and to conduct a comprehensive review of the related literature.

In a China-based study conducted in 2021, the effectiveness of diffusion tensor imaging (DTI) in diagnosing contrast-induced acute kidney injury was investigated using animal models. A total of 45 rats were divided into three groups: One group received contrast media on both days, another received contrast media on the first day and saline on the second, and the control group received saline on both days. MRI was performed on randomly selected rats (n = 5 per group) at three time points: one, 24, and 120 hours. DTI parameters, including fractional anisotropy and ADC values, were analyzed and compared to histopathological findings and hypoxia-inducible factor 1-alpha (HIF-1α) antibody expression to assess renal injury. The results showed that ADC values, particularly in the renal medulla, demonstrated a moderate correlation with histopathological findings and a strong correlation with HIF-1α expression. Accordingly, the study concluded that MRI may effectively detect acute kidney injury [17].

In another study from China, Duan et al. examined the utility of ADC differences measured using DWI in evaluating chronic kidney disease in patients with type 2 diabetes. Their findings demonstrated that ADC values correlated with histopathological data, supporting DWI as a reliable and non-invasive biomarker for assessing renal function [18].

In a study conducted by Ferguson et al., DWI and histopathological findings were compared in patients with renal damage secondary to renovascular disease. The results demonstrated that DWI was effective in detecting renal fibrosis and associated kidney injury, highlighting its potential role in the non-invasive assessment of parenchymal damage [19].

In a 2018 meta-analysis by Liu et al., the effectiveness of DWI in staging kidney injury was evaluated through a comprehensive literature review. Out of 146 identified studies, six met the inclusion criteria for the meta-analysis. The findings indicated that DWI is a useful non-invasive modality for assessing renal function and detecting early kidney injury. However, limitations were noted due to the small number of included studies, heterogeneous sample sizes, and methodological differences, such as variations in imaging devices and calculation techniques, which may have impacted the overall conclusions of the meta-analysis [20].

According to the current literature, utilizing DWI to assess renal injury, several investigations have explored the use of DWI as a non-invasive alternative to invasive diagnostic methods for detecting early damage related to AR following kidney transplantation.

In an animal study conducted by Yang et al., DWI was performed using a 7 Tesla MRI scanner on 29 rats, including 20 healthy controls and nine transplant recipients. On postoperative day 4, a decrease in graft ADC values was observed in the transplant group, suggesting that DWI may be effective in identifying graft injury secondary to AR [21].

In another study by Thoeny et al., DWI was conducted in 15 kidney transplant recipients without complications and 15 healthy volunteers to evaluate renal function. No significant differences were observed in the cortical and medullary ADC values of the renal allografts. However, despite the lack of statistical significance, focal hyperintense regions on histopathological examination corresponded to areas with reduced ADC values, suggesting a potential association with AR [22].

In a Germany-based prospective study evaluating renal allograft function using DTI, 40 transplant recipients were analyzed. The results demonstrated that both cortical and medullary ADC values were correlated with eGFR, indicating that ADC mapping through diffusion MRI is an effective method for assessing renal function posttransplantation [23].

In our study, allograft function following kidney transplantation was clinically assessed using eGFR and urinary protein levels. It was observed that the study group with AR or ATN had lower eGFR values and higher urinary protein levels compared to the control group. These findings suggest that biochemical markers are effective in reflecting allograft function.

ADC values measured by DWI were significantly lower in all regions of the transplanted kidneys in the study group than in the control group, indicating a correlation with reduced renal function. Our results demonstrated that decreased ADC values corresponded with reductions in renal function and were consistent with biochemical markers, supporting the utility of DWI in detecting renal injury.

A literature review shows that ADC measurements often yield consistent results in both the renal cortex and medulla. In our study, when ADC values were compared across different anatomical regions, they were significantly different in all areas except for the upper pole of the cortex and medulla, highlighting the regional diagnostic performance of DWI.

Beyond the diagnosis of renal injury, several studies have demonstrated the potential use of DWI in monitoring allograft function over time. In a study conducted by Kaul et al. in 2014, ADC values of transplanted kidneys on postoperative day 7 were compared to biopsy results. It was observed that ADC values decreased in both the cortex and medulla of the kidneys, exhibiting functional impairment. Moreover, an increase in ADC values was detected in patients who had received treatment for AR and showed clinical improvement, suggesting that DWI may also serve as a valuable tool in monitoring treatment response [24].

In the multicenter, randomized controlled ZEUS trial conducted by Mani et al., the impact of immunosuppressive therapy on allograft function was evaluated using ADC measurements obtained through DWI. The study compared patients receiving cyclosporine A to those switched to everolimus. The results indicated that patients on cyclosporine A exhibited lower ADC values, whereas those switched to everolimus demonstrated increased ADC values in parallel with improvements in eGFR. These findings support the potential utility of DWI in the follow-up of renal allograft function [25].

This study has several limitations. First, the sample size was relatively small, which may limit the generalizability of the findings. Second, the diagnoses of AR and ATN were made based on clinical evaluation, and no histopathological confirmation was obtained for this study. This lack of biopsy data prevented a direct comparison of DWI findings with definitive histopathological diagnoses.

## Conclusions

In conclusion, this study highlights the potential utility of DWI as a non-invasive tool for assessing renal allograft function and detecting early graft injury in kidney transplant recipients. Although histopathological confirmation was not available, our findings are consistent with previous research demonstrating the diagnostic and monitoring value of DWI. Larger studies are needed to further establish DWI as a reliable adjunct in clinical decision-making for graft surveillance and the early detection of complications.
